# Time-Dependent Effect of Encapsulating Alginate Hydrogel on Neurogenic Potential

**DOI:** 10.22074/cellj.2016.3736

**Published:** 2015-07-11

**Authors:** Shahnaz Razavi, Zahra Khosravizadeh, Hamid Bahramian, Mohammad Kazemi

**Affiliations:** 1 Department of Anatomical Sciences and Molecular Biology, School of Medicine, Isfahan University of Medical Sciences, Isfahan, Iran; 2 Department of Genetic, School of Medicine, Isfahan University of Medical Sciences, Isfahan, Iran

**Keywords:** Alginate, Mesenchymal Stem Cells, Neurogenic Differentiation, Proliferation, Tissue Engineering

## Abstract

**Objective:**

Due to the restricted potential of neural stem cells for regeneration of central nervous system (CNS) after injury, providing an alternative source for neural stem cells is essential. Adipose derived stem cells (ADSCs) are multipotent cells with properties suitable for tissue engineering. In addition, alginate hydrogel is a biocompatible polysaccharide polymer that has been used to encapsulate many types of cells. The aim of this study was to assess the proliferation rate and level of expression of neural markers; *NESTIN*, glial fibrillary acidic protein (*GFAP*) and microtubule-associated protein 2 (*MAP2*) in encapsulated human ADSCs (hADSCs) 10 and14 days after neural induction.

**Materials and Methods:**

In this experimental study, ADSCs isolated from human were cultured in neural induction media and seeded into alginate hydrogel. The rate of proliferation and differentiation of encapsulated cells were evaluated by 3-[4, 5-dimethylthiazol-2-yl]-2, 5-diphenyl tetrazolium bromide (MTT) assay, immunocytoflourescent and realtime reverse transcriptase polymerase chain reaction (RT-PCR) analyzes 10 and 14 days after induction.

**Results:**

The rate of proliferation of encapsulated cells was not significantly changed with time passage. The expression of *NESTIN* and *GFAP* significantly decreased on day 14 relative to day 10 (P<0.001) but *MAP2* expression was increased.

**Conclusion:**

Alginate hydrogel can promote the neural differentiation of encapsulated hADSCs with time passage.

## Introduction

Nerve injuries and neurodegenerative diseases are comparatively common clinical problems that often lead to persistent sensory and motor impairments in patients (1). Tissue engineering tries to provide biological replacements from specific cells and polymeric scaffolds for treatment of damaged tissues (2).

Because embryonic stem cells have histocompatibility and ethical limitations, mesenchymal stem cells such as human adipose derived stem cells (hADSCs) are one of the promising keys to success in the treatment of neurologic disorders (3-5). Adipose tissue is harvested by less invasive procedures as an alternative source of multipotent stromal cells which gives access to an abundant quantity of stem cells (5-7). hADSCs have the capacity of multi-lineage differentiation such as chondrocytes, osteoblasts, adipocytes, myocytes and neuron-like cells *in vitro* under particular conditions (7-11). The stem cell quantity extracted from adipose tissue is higher than those of bone marrow tissue (2 vs. 0.002%) (12). In addition, neurospecific trophins, metabolic genes and neuroprotective molecules are expressed by hADSCs (5, 13, 14).

Hydrogels can serve as biocompatible scaffolds that provide appropriate structure to controlled drug delivery to tissues and cultures, and serve as adhesives or barriers between tissue and material surfaces (15). Alginate hydrogel is a water-soluble natural polysaccharide consisting of 1-4 Linked ß-D-mannuronic acid (M) and α-L guluronic acid (G) monomers (16-18). During gel-formation, high-G gels show high porosity and low shrinkage, however, high-M gels become softer and more elastic, and their porosity is decreased (19).

Since central nervous system (CNS) represents an immunologically privileged site, alginate-encapsulated cells may well be endured (20). Encapsulated cells in alginate hydrogel does not cause immune response because pure alginate beads persuade the same immunological reaction (21). Alginate polysaccharide sequences might imitate functional groups within the extracellular matrix of the brain, which can adjust signal transduction cascades to guide cell migration and neurite growth (22).

Generating neuron-like cells from stem cells at a high rate could be useful for treatment of nerve injuries. However, it has not yet been evaluated whether time passage has a positive or negative effect on the rate of neural differentiation of encapsulated hADSCs in alginate hydrogel. Therefore, in the present study, proliferation rate and level of expression of neural markers, *NESTIN* (as neural precursor marker), glial fibrillary acidic protein (*GFAP*, as glial marker) and microtubule-associated protein 2 (MAP2, as mature neuron marker) in encapsulated hADSCs were assessed by real-time reverse transcriptase polymerase chain reaction (RT-PCR) and immunocytoflourescent analyzes10 and14 days after neural induction.

## Materials and Methods

### Human adipose derived stem cells isolation and culture

In this experimental study, hADSCs were isolated from subcutaneous adipose tissue of 3 female donors during abdominal surgery upon gaining written consent approved by Care Committee of Isfahan University of Medical Sciences. Human ADSCs were cultured according to a previous study (23). Adipose tissue was washed three times by sterile phosphate buffer saline (PBS, Gibco, BRL, Paisley, UK) to eliminate red blood cells and debris. Samples were digested by 0.01% collagenase type I (Sigma, St. Louis, Mo, USA) for 30 minutes at 37˚C. After neutralization of the enzyme with the same volume of Dulbecco’s modified Eagles medium (DMEM-F12, PAA Laboratories GmbH, Austria) containing 10% fetal bovine serum (FBS, Gibco BRL, Paisley, UK), the cell suspension was centrifuged for 10 minutes at 1600 rpm. The cell pellet was suspended in DMEM-F12, supplemented by 10% FBS and 1% penicillin/streptomycin (Gibco, BRL, Paisley, UK), and incubated at 37˚C and 5% CO_2_. After cells reached nearly 90% confluency, they were trypsinized and subcultured. Human ADSCs for this study were used at passage 3-5.

### Characterization of human adipose derived stem cells

In order to determine "stemness" of isolated cells, human ADSCs within 3-5 passages were harvested by trypsinization and then washed twice with 1% bovine serum albumin (BSA)/PBS (Gibco, BRL, Paisley, UK) and incubated with antibodies against cluster of differentiation 90 (CD90), CD44, CD105, CD34, CD14 and CD45 for 30 minutes. Primary antibodies were directly conjugated with fluorescein isothiocyanate (FITC) or Phycoerythrin (Chemicon, Temecula, CA, USA). For isotype control, non-specific FITC-conjugated IgG was substituted for the primary antibodies. Flow cytometry was performed usinga FACscanflow cytometry (Becton Dickinson, San Jose, CA).

### Induction of neurogenic differentiation

The isolated cells were dissociated by 0.25% trypsin-EDTA (Gibco, BRL, Paisley, UK) and counted hADSCs were placed on low-attachment plastic tissue culture plates at a concentration of 1×10^6^ in DMEM-F12 supplemented with 2% B27, 20 ng/ml basic fibroblast growth factor (bFGF, Gibco, BRL, Paisley, UK), 20 ng/ml human epidermal growth factor (hEGF, Gibco, BRL, Paisley, UK) and 2 μl heparin (Sigma, St.Louis, MO, USA). Growth factors and supplements were added twice every 3 to 4 days. After neurospheres were formed, they were singled by 0.25% Trypsin-EDTA. For terminal differentiation, a portion of singled neurosphere cells were encapsulated in alginate hydrogel and other portion of singled cells as control were plated in 24-well plate in neurobasal medium supplemented by 5% FBS, 1% penicillin/ streptomycin, 1% L- glutamine , 1% N2, 1% nonessential amino acids, 2% B27 and 1% Nystatine for 7 days. All growth factors and supplements, except where specified otherwise, were purchased from Gibco BRL, Paisley, UK.

### Encapsulation of singled neurospheres in alginate hydrogel

Alginic acid sodium salt (Sigma, St.Louis, MO, USA) was dissolved in sodium chloride (Sigma, St.Louis, MO, USA) (0.9% w/v) and filtered to obtain a 1.2% alginate solution. Singled neurospheres were then re-suspended at 1×10^6^/ml in sterile sodium alginate and dropped by a 22-gauge needle in to a 102 mM CaCl_2_ (Sigma, St.Louis, MO, USA) solution.

The suspension was kept for 1 hour at room temperature to form alginate beads. The solution was removed and beads were rinsed with PBS twice and once with DMEM-F12 medium. Neural induction medium was then added to the plate containing alginate encapsulated cells. Prepared beads were finally incubated at 37˚C and 5% CO_2_. All examinations were done 10 and 14 days after neural induction.

### 3-[4, 5-dimethylthiazol-2-yl]-2,5-diphenyl tetrazolium bromide (MTT) assay

In order to determine the effect of encapsuling cells in alginate hydrogelon cell viability with time passage, alginate beads (25×10^3^ cells/well) were seeded into each well of 24-well plates for 10 and 14 days.

Neural induction medium of each well was aspirated and 200 μl of DMEM-F12 along with 20 μl of MTT solution was then added. The cell-cultured plates were incubated at 37˚C in 5% CO_2_ for 4 hours. The supernatant was discarded and 200 μl of dimethyl sulfoxide (DMSO, Sigma, St.Louis, MO, USA) was added. After pipetting of the DMSO solution, the absorbance of each well was determined by a microplate reader (Hiperion MPR 4^+^, Germany) at the wave length of 540 nm.

### Morphology observation

The morphology of alignate-encapsulated cells was assessed by scanning electron microscopy (SEM, Seron Technology AIS 2500, India). Beads were fixed in 4% paraformaldehyde (Sigma, St.Louis, MO, USA) and frozen sections were prepared (cryocut1800, reichert, JUNG, Germany). Thin sections of the cellseeded alginate were gold-sputtered and examined by SEM (Seron Technology AIS 2500, India).

### Immunocytoflourescent analysis

Differentiated cells in alginate beads were fixed in 4% paraformaldehyde and 70% ethanol for 30 minutes. Samples were then permeabilized with 2% Triton X-100 (Sigma, St.Louis, MO, USA) for 30 minutes. Blocking in 1 mg/ml BSA and incubating primary antibodies against mouse anti-NESTIN (1:300, Abcam, Cambridge, MA, USA), mouse anti- GFAP (1:600, Abcam, Cambridge, MA, USA) and mouse anti-MAP2 (1:300, Abcam, Cambridge, MA, USA) were performed overnight. The secondary antibody, anti-mouse FITC-conjugated IgG antibody (1:500, Abcam, Cambridge, MA, USA), was used for 2 hours at 37˚C. For nucleus visualization, cells were stained with diamidino-2-phenylindole (DAPI, 1:1000, Sigma, St.Louis, MO, USA). For negative control, primary antibody was eliminated. To merge the pictures, image J software1.42 software (Wayne Rasband, National Institutes of Health, Bethesda, MD, USA) was used. Hundred cells were counted per sample.

### Real-time reverse transcriptase polymerase chain reaction (RT-PCR) analysis

To release encapsulated induced cells, the alginate beads were incubated in a solution containing 15 mM sodium citrate (Sigma, St.Louis, MO, USA) and 150 mM NaCl (Sigma, St.Louis, MO, USA). Total RNA was isolated from encapsulated cells using RNeasy mini RNA isolation kit (Qiagene, Hilden, Germany) according to the manufacturer’s protocols. After, cDNA was synthesized using total RNA, oligo-dT, primers and reverse-transcriptase (Fermentas, GMBH, Germany). The real-time PCR was performed with gene specific primers and the SYBRGreen PCR Master Mix (Qiagene, Hilden, Germany) using a thermal cycler rotor-gene 6000 (Qiagene, Hilden, Germany). The primer sequences are shown in table 1. The gene of interest was normalized against the reference gene *glyceraldehydes-3-phosphate dehydrogenase* (*GAPDH*). The expression level of each target gene was calculated by 2^-ΔΔCT^.

**Table 1:** The primer sequences (forward, reverse) used in real time reverse transcriptase polymerase chain reaction analysis


Gene	Forward (top) Reverse (bottom)

*NESTIN*	5´-AACAGCGACGGAGGTCTCTA-3´
	5´-TTCTCTTGTCCCGCAGACTT-3΄
*MAP2*	5´-TCAGAGGCAATGACCTTACC-3´
	5´-GTGGTAGGCTCTTGGTCTTT-3´
*GFAP*	5´-CCTCTCCCTGGCTCGAATG-3´
	5´GGAAGCGAACCTTCTCGATGTA-3΄
*GAPDH*	5´-ACCACAGTCCATGCCATCAC-3´
	5´-TCCACCACCCTGTTGCTGTA-3΄


### Statistical analysis

Data obtained from MTT, immunocytoflourescent and real-time RT-PCR assays were analyzed by one-way ANOVA. Data were expressed as mean ± standard error (SE). Statistical significance was considered when P<0.05.

## Results

### Morphological features of human adipose derived stem cells during culture and neural induction

**Fig.1:**
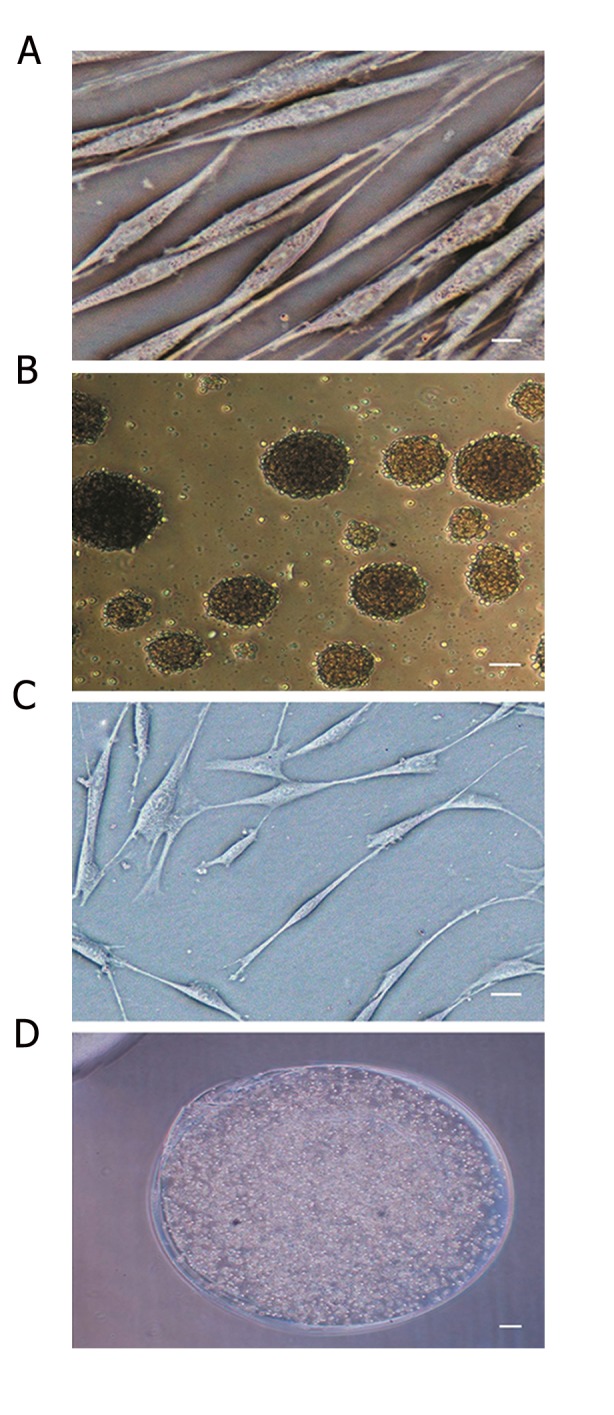
Morphological characteristics of human adipose derived stem cells (hADSCs) following neural induction and encapsulation in alginate hydrogel. A. Undifferentiated hADSCs cultured in Dulbecco’s modified Eagles medium (DMEM-F12) exhibited a fibroblastic morphology, B. hADSCs cultured for 7 days in neural induction medium (neurospheres were observed), C. Induced hADSCs showed cytoplasmic retraction and ramified shapes and D. Encapsulated hADSCs in alginate hydrogel. Scale bars in A and B is 200 μm and in C and D is 50 μm. Samples (n=3), experiments (n=3), replicates (n=3).

The isolated hADSCs were observed by phase contrast microscopy throughout culture and differentiation. They presented a mono-layer of large and spindle-shaped cells resembling fibroblast cells after 2 passages (Fig.1A). Flow Cytometric analysis showed that more than 90% of the isolated hADSCs expressed mesenchymal stem cells (MSC)-specific markers, including CD105, CD44 and CD90, but less than 1% of the isolated hADSCs expressed markers for hematopoietic stem cells or endothelial cells, including CD14, CD45 and CD34. Thus, in this experiment hADSCs appeared to be MSCs.

**Fig.2:**
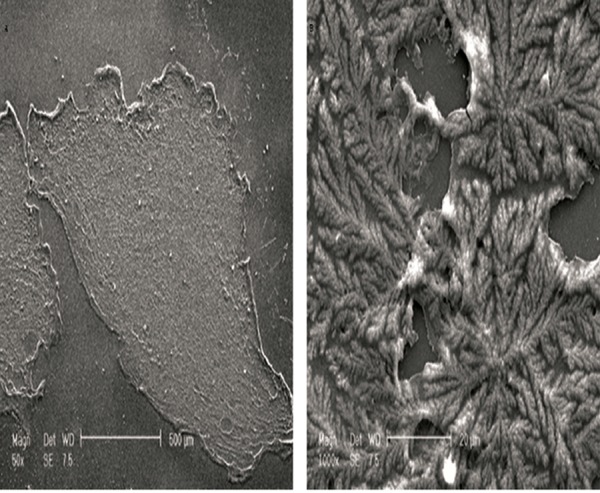
Scaning electron micrograph of a crayo-section of induced human adipose derived stem cells (hADSCs) within an alginate bead shows a mossy-like network of alginate hydrogels containing spherical cells. Arrows point to spherical cells. Samples (n=3), experiments (n=3) and replicates (n=3).

After culture in neural induction medium, hADSCs and neurosphere formation exhibited cytoplasm retraction and a spherical cell body appearance with multiple cell processes, thus showing a neural appearance (Fig.1B, C), while encapsulated cells in alginate had round appearance (Fig.1D). The SEM micrograph of alginate bead indicated a network structure and induced cells in the alginate networks had spheroid shapes (Fig.2).

### Cell viability

**Fig.3:**
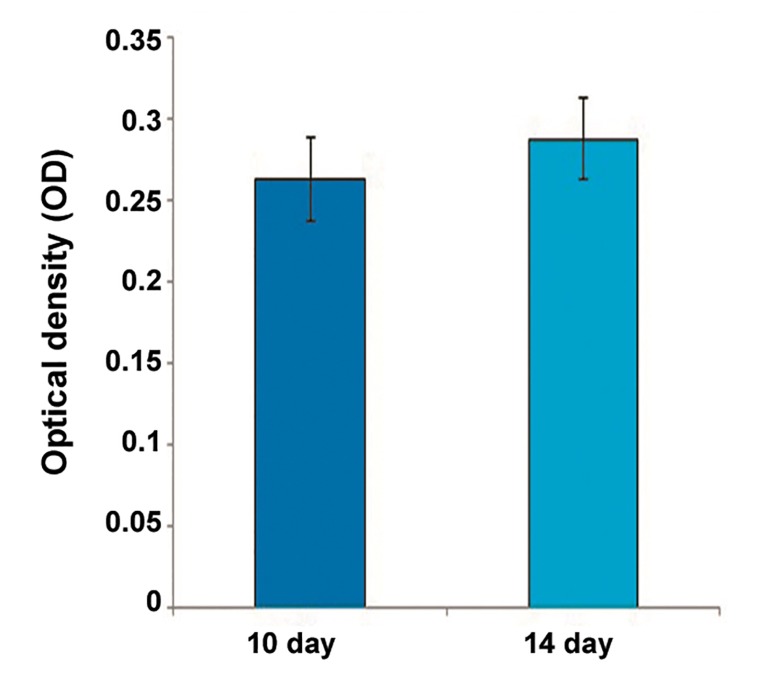
Optical density (OD) determination for encapsulated cells at 540 nm, 10 and 14 days post induction. The mean OD of encapsulated cells was not significantly different between days 10 and 14. Values are mean ± standard error (SE).

Survival of differentiated hADSCs in alginate beads was determined at 10 and 14 days after induction. The mean optical density (OD) of encapsulated cells was not significantly different between days 10 and 14 (0.26 ± 0.02 vs. 0.28 ± 0.02) (Fig.3).

### Immunocytoflourescence after encapsulation of induced hADSCs in alginate hydrogel

Ten and fourteen days after neural differentiation, encapsulated cells in alginate hydrogel were labeled with NESTIN, GFAP and MAP2 and cell nuclei were counterstained with DAPI.

**Fig.4:**
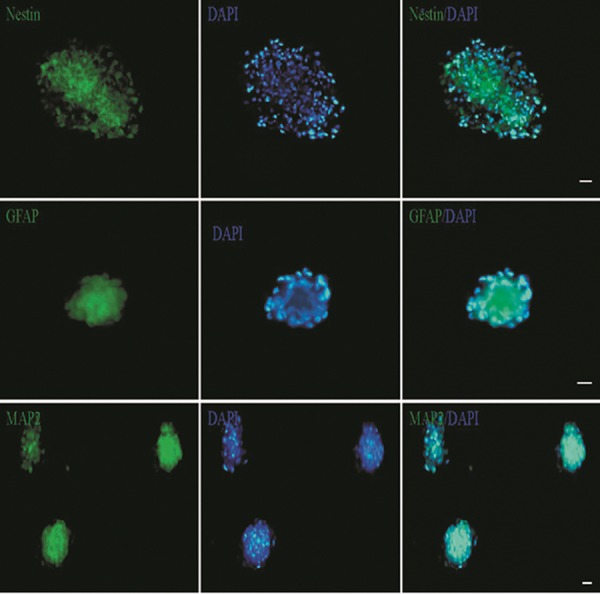
Immunocytoflourescent staining for neural markers [NESTIN, glial fibrillary acidic protein (GFAP) and microtubule-associated protein 2(MAP2)] in encapsulated human adipose derived stem cells (hADSCs) at 14 days after induction. All nuclei were counterstained with diamidino-2-phenylindole (DAPI). Scale bar: 50 μm. Samples (n=3), experiments (n=3) and replicates (n=3).

The mean percentage of positive cells for neural markers NESTIN (progenitor neural), GFAP (astrocyte) and MAP2 (mature neural) was evaluated at 10 and 14 days after induction (Fig.4).

Immunocytoflourescent analysis showed that the mean percentage of NESTIN in encapsulated cells at day 14 was increased (91.90 ± 1.84%) compared with that at day 10 (72.2 ± 0.80), while the mean percentage of GFAP decreased on day 14 (56.75 ± 7.30%) compared with that on day 10 (65.66 ± 2.33%) after induction.

**Fig.5:**
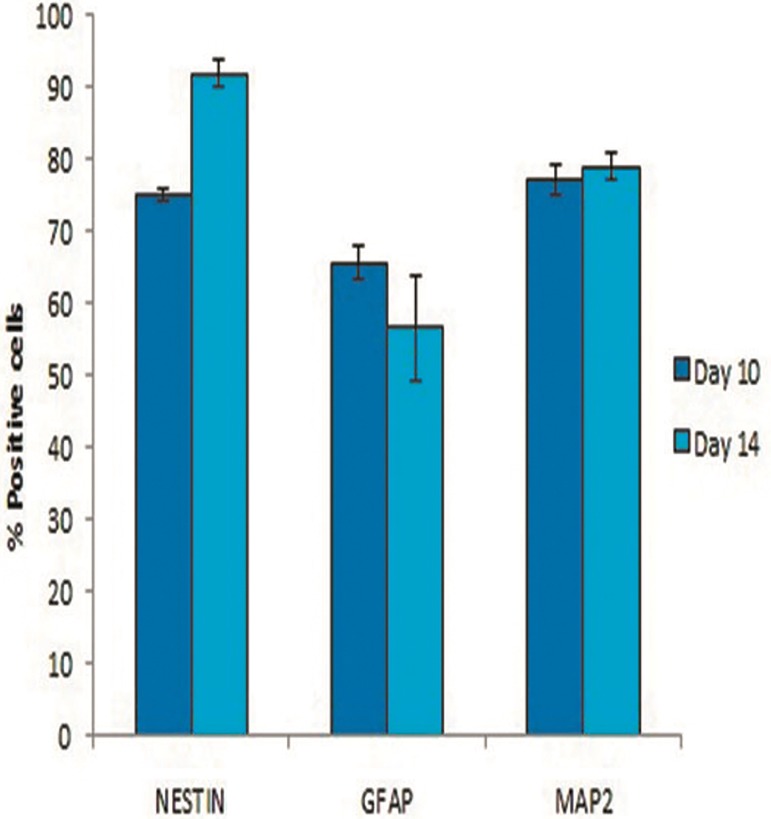
Comparison of mean positive cells for NESTIN, glial fibrillary acidic protein (GFAP) and microtubule-associated protein 2 (MAP2) markers in encapsulated cells 10 and 14 days after induction. No significant difference was observed. The positive rates were shown as mean ± standard error (SE). Samples (n=3), experiments (n=3) and replicates (n=3).

Similar to NESTIN, the mean percentage of MAP2 increased from day 10 (77.23 ± 2.20%) to day 14 (78.96 ± 1.81%) after induction (Fig.5).

### Real-time reverse transcriptase polymerase chain reaction analysis

In order to determine the effect of alginate hydrogel on the expression of neural markers in induced hADSCs, real-time RT-PCR analysis was performed.

**Fig.6:**
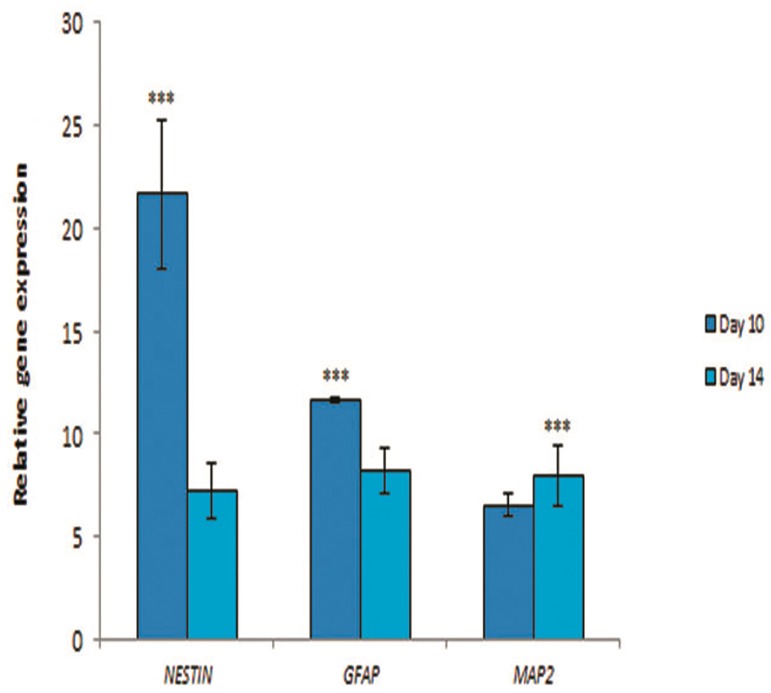
Real-time reverse transcriptase polymerase chain reaction (RT-PCR) analysis of the encapsulated cells 10 and 14 days after induction. The expression of *NESTIN* and glial fibrillary acidic protein (*GFAP*) in encapsulated human adipose derived stem cells (hADSCs) at day 14 was significantly down-regulated compared with day 10, while the expression of microtubule-associated protein 2 (*MAP2*) in encapsulated hADSCs at day 14 was significantly up-regulated relative to day 10 . Values are mean ± standard error (SE). ***; P<0.001. Samples (n=3), experiments (n=3) and replicates (n=3).

The level of expression of *NESTIN* in encapsulated cells at day 14 was significantly down-regulated (7.22 ± 1.36) compared with encapsulated cells at day 10 (21.67 ± 3.60, P<0.001). Also the results of real-time RT-PCR analysis showed that *GFAP* expression in encapsulated cells at day 14 was significantly down-regulated (8.26 ± 1.11) compared with encapsulated cells at day 10 (11.64 ± 0.10, P<0.001). Moreover, the level of expression of MAP2 in encapsulated cells at day 14 was significantly up-regulated (7.93 ± 1.45) relative to encapsulated cells at day 10 (6.55 ± 0.6, P<0.001, Fig.6).

## Discussion

Our study demonstrates that induced cells in alginate beads can promote differentiation of hADSCs. In addition, the MTT assay showed that the proliferation of hADSCs was increased in alginate hydrogel with time passage.

Our results show that expression of *NESTIN* and *GFAP* on day 14 was significantly decreased compared with expression of these markers on day 10, while *MAP2* expression was significantly up-regulated with time passage. Consistent with real-time RT-PCR results, immunocytoflourescentanalysis showed that the mean percentage of GFAP was decreased while otherwise for NESTIN and MAP2.

Some evidence show that many cell types encapsulated in alginate hydrogel have limited cell proliferation (24-26). The cell proliferation was decreased in alginate culture, which may be related to the cell death rise during encapsulation by a temporary reaction to the toxicity of CaCl_2_ (24) and relatively low alginate weight percentage (1%) used (27). Also, the proliferation of MSCs is anchorage-dependent and alginate hydrogel, by procuring a suspension condition, can "synchronize" and stop cells in G0-G1 phase (28).

Previous study have demonstrated that hADSCs express a range of neurotrophic factors such as nerve growth factor (NGF), brain derived neurotrophic factor (BDNF) and glial cell derived neurotrophic factor (GDNF) (5). Laminin, the important extracellular matrix (ECM) molecule for nerve regeneration is expressed by hADSCs (29). Moreover, vascular endothelial growth factor (VEGF), which is expressed by hADSCs, can promote neurite outgrowth (30, 31).

Purcell et al. (27) indicated that cortical neural stem cells (NSCs) encapsulated in alginate secreted BDNF on day 4. Also encapsulated NSCs expressed NESTIN and GFAP. In addition, we showed that ADSCs release BDNF, GDNF and NGF (23). BDNF has many roles in brain development, adult neuroplasticity, neural survival, neurogenesis, neurite outgrowth and synaptic plasticity (32, 33) and also increases neurogenesis and promotes the differentiation and survival of newly generated neurons (34).

Banerjee et al. (35) reported that NSCs encapsulated in alginate expressed the greatest enhancement of the neural marker β-tubulin ΙΙΙ within the softest hydrogel after 7 days of culture.

However, Matyash et al. (36) showed that no functionalized, soft alginate hydrogels, formed by crosslinking with Ca^2+^, supported fast and plentiful neurite growth from neurons in primary rat neuronal cultures.

Studies have indicated that increase in hydrogel stiffness causes decreased permeability and subsequent decrease in viability and proliferation of NSCs encapsulated in it (35). Our results are consistent with previously published reports which demonstrated that neurons prefer soft rather than stiff states but its mechanism is not yet known.

## Conclusion

Overall, we demonstrate that alginate hydrogel influences viability and neural differentiation of hADSCs with time passage. The viability of encapsulated hADSCs non significantly increased with time, however, encapsulation promoted neural differentiation. It may be possible that hADSCs encapsulated in alginate hydrogel secrete neurotrophic factors to promote neural differentiation. Identification of the molecular mechanisms of neural differentiation and quantification of neurotrophic factors released from hADSCs encapsulated in alginate hydrogel, could provide valuable information for applications in tissue engineering and *in vivo* studies.
